# THE STATE OF THE DIGITAL DIVIDE

**DOI:** 10.1093/geroni/igad104.0818

**Published:** 2023-12-21

**Authors:** Walter Boot

## Abstract

Over the past few decades, the age-related digital divide has narrowed. For example, since the year 2000 in the United States, the percentage of older adults (65+) online has increased dramatically from 14% to 75%. However, compared to the 96% of individuals 50 to 64 years of age online, this still represents a substantial divide. Similar trends are observed in terms of social media usage and smartphone adoption, and even for older adults who own a smartphone, on average, smartphone proficiency can be substantially lower relative to younger people. This divide can put older adults at a substantial disadvantage and lock them out of the many benefits of existing and emerging technologies. This session will tackle issues surrounding the age-related digital divide. S. Cotten will provide a broad overview of the digital divide and theoretical perspectives that can help explain it. J. Chung will describe how the digital divide impacted social connectivity during the pandemic in low-income communities. S. Pace will discuss African American older adults, ICT use, and loneliness. S. Zhang will discuss attitudinal contributions to technology use and adoption relevant to the digital divide. Finally, W. Boot will discuss technology access and adoption and intersectionality and will discuss potential solutions to close or eliminate the gap in technology adoption and proficiency between younger and older people.

